# Rediscover the predictive capacity of B-type natriuretic peptide applied to neonatal supraventricular tachycardia

**DOI:** 10.1186/s12872-023-03646-5

**Published:** 2023-12-08

**Authors:** Yaheng Lu, Ying Xiong, Yizhou Wen, Yanfeng Yang, Hanmin Liu

**Affiliations:** 1 Department of Pediatrics, West China Second University Hospital, Sichuan University, Chengdu, China; 2 Key Laboratory of Birth Defects and Related Diseases of Women and Children (Sichuan University), Ministry of Education, Chengdu, 610041 China; 3 Department of Pediatric Cardiology, School of Medicine, Chengdu Women’s and Children’s Central Hospital, University of Electronic Science and Technology of China, Chengdu, Sichuan, 611731 China; 4 Key Laboratory of Chronobiology (Sichuan University), National Health Commission of China, Chengdu, China

**Keywords:** Supraventricular tachycardia, Acute heart failure, B-type natriuretic peptide, Anti-arrhythmic treatment, Prediction, Neonates

## Abstract

**Background:**

Supraventricular tachycardia (SVT) is one of the most common non-benign arrhythmias in neonates, potentially leading to cardiac decompensation. This study investigated the early risk factors of acute heart failure (AHF) secondary to SVT in neonates, and explored their value in guiding the selection of effective anti-arrhythmic treatment.

**Methods:**

A total of 43 newborns diagnosed with and treated for SVT between January 2017 and December 2022 were analyzed. According to the presence of AHF after restoring sinus rhythm in newborns with SVT, they were divided into SVT with AHF group and SVT without AHF group. Clinical data and anti-arrhythmic therapies were analyzed. Risk factors of AHF secondary to SVT in neonates were determined using logistic regression. The cut-off value for predictors of AHF secondary to SVT and demanding of a second-line anti-arrhythmic treatment was determined through receiver operating characteristic (ROC) analysis.

**Results:**

Time to initial control of tachycardia > 24 h, hyperkalemia, anemia, and plasma B-type natriuretic peptide (BNP) were identified as risk factors of AHF secondary to SVT in neonates. BNP exhibited AUC of 0.80 in predicting AHF, and BNP > 2460.5pg/ml (OR 2.28, 95% CI 1.27 ~ 45.39, *P* = 0.03) was an independent predictor, yielding sensitivity of 70.6% and specificity of 84.6%. Neonates with BNP > 2460.5pg/ml (37.5% versus 7.4%, *P* = 0.04) had a higher demand for a second line anti-arrhythmic treatment to terminate SVT, with sensitivity and specificity for BNP in predicting at 75.0%, 71.4%, respectively.

**Conclusions:**

BNP could be used to predict an incident of AHF secondary to SVT and a demand of second-line anti-arrhythmic treatment to promptly terminate SVT and prevent decompensation in neonates.

## Introduction

Supraventricular tachycardia (SVT), one of the most common neonatal arrhythmias, is often asymptomatic and rarely life-threatening [1]. However, a small number of newborns may develop signs of congestive heart failure and cardiogenic shock either before or after anti-arrhythmic therapy [2]. Prolonged or intermittent recurrence of SVT may be linked to the immaturity of the conduction system in neonates. Additionally, unstable prenatal and postnatal states may trigger this type of arrhythmia [3–5]. Factors affecting the overall cardiovascular status could collectively contribute to hemodynamic instability, resulting in acute heart failure (AHF) secondary to SVT in neonates, thereby increasing the mortality rates and prolonging hospitalization [6].

The optimal protocol for terminating SVT, considering both safety and efficacy, has not been established. However, the primary objective of promptly restoring sinus rhythm is crucial in preventing cardiac decompensation and mortality [7, 8]. Early recognition of AHF before the onset of obvious signs and symptoms could expedite treatment and help prevent a poor prognosis. Our retrospective study evaluated the early risk factors of AHF secondary to SVT in neonates and preliminary explored their role in the selection of anti-arrhythmic treatment.

## Methods

Patients were identified by retrospective analysis of the database at Chengdu Women’s and Children’s Central Hospital, School of Medicine, University of Electronic Science and Technology of China (UESTC) from January 2017 to December 2022. Our study included all newborns ≤ 28days of age who were diagnosed with SVT and received treatment in the Neonatal Intensive Care Unit (NICU). Excluded from this study were newborns with the following conditions: presence of AHF prior to the onset of SVT, SVT following cardiac surgery, and inability to determine the specific onset time of SVT.

Data on acute heart failure, including primary variables of interest, were reviewed for each patient. Additionally, information on age at SVT diagnosis, sex, birth weight, time to initial control of tachycardia, prematurity, birth asphyxia, elderly parturient, fetal distress, prenatal history of tachycardia, caesarean section, maternal systemic disease, plasma B-type natriuretic peptide (BNP) levels, and comorbid conditions such as congenital heart disease (CHD), electrolyte disorders, systemic infection, hypoproteinemia, anemia, acidosis, hypoxemia, and anti-arrhythmic treatment (including pharmacological and non-pharmacological procedures), were also reviewed.

All newborns included in the study were transferred to NICU from the standard ward. The onset of SVT occurred within 24 h of admission. Initial control of tachycardia was defined as no recurrence of SVT within 24 h. As preserved left ventricular function, measured by echocardiography in all included neonates, heart failure was defined as a sum of clinical score ≥ 3, according to a modified Ross scoring system (range: 0–12 points), within 6 h after the final termination of SVT to avoid any disturbance caused by SVT on heart rate [9]. Prematurity was defined as a gestational age < 37 weeks. Birth asphyxia (BA) was defined as a failure to initiate spontaneous respiration and/or a 5-minute Apgar score < 7 [10]. Anemia was defined as a hemoglobin (Hb) or hematocrit concentration of > 2 standard deviations below the mean for postnatal age [11]. Acidosis and hyoxemia were defined as pH ≤ 7.2 and PaO_2_ ≤ 50mmHg respectively. Hypoproteinemia was defined as serum albumin < 30 g/L. The normal level of sodium, potassium and ionized calcium was taken as 130–145 mmol/L, 3.7–5.9 mmol/L and 1-1.5 mmol/L, respectively. Fetal distress was diagnosed based on electronic monitoring of the fetal heart, counting fetal movements, and assessing the characteristics of the amniotic fluid by obstetrician [12]. The level of plasma B-type natriuretic peptide (BNP) should be measured on admission prior to anti-arrhythmic treatment. The normal reference value for children is 0-500ng/ mL, but this range may not be applicable to neonates.

The type of tachycardia was assessed based on the surface electrocardiogram (ECG) and broadly classified SVT into two subgroups: re-entry or automatic tachycardias [13]. The selection of anti-arrhythmic drugs and doses was based on echocardiography and the preference of attending physician, following international consensus statement. In general, the Vagal Maneuver (diving reflex) was applied to all patients by using ice-cold water on their faces, Adenosine was administered for re-entry tachycardias, while digitalis and beta-blockers (esmolol) were used for automatic tachycardias. These treatments were classified as first-line treatment in our study, as reported [14]. Intravenous propafenone was used for long-standing, recurrent SVT if there were no signs of heart failure. Amiodarone was the treatment of choice in the presence of heart failure. DC cardioversion was performed when there was hemodynamic instability or when drug cardioversion was ineffective. Despite their attractive efficacy, these treatments were reserved as second-line therapy in cases of refractory of SVT, due to their relatively high incidence of systemic adverse effects [2, 15].

This retrospective study was approved by the Ethics Committee of Chengdu Women’s and Children’s Central Hospital, School of Medicine, UESTC, and in accordance with the 1964 Helsinki declaration and its later amendments or comparable ethical standards. The requirement for informed patient consent was waived.

## Statistical analysis

Continuous variables are presented as mean ± standard deviation. Categorical variables are presented as frequency with percentage. Univariate and multivariate logistic regression analyses were performed to identify risk factors for AHF secondary to SVT in neonates. Receiver operating characteristic curve (ROC) analysis for predictors of AHF secondary to SVT and demanding of a second line anti-arrhythmic treatment was performed. Fisher’s exact test was used to compare categorical variables. Statistical significance was defined as a *P* < 0.05.

## Results

A total of 43 newborns diagnosed SVT—17 boys and 26 girls, aged from 0 to 27 days old— met the inclusion criteria. Of these patients, 17 newborns who developed AHF secondary to SVT were assigned to SVT with AHF group, while the other 26 patients were assigned to SVT without AHF group.

A history of prenatal tachyarrhythmia was present in 12 patients (27.9%), while fetal distress was present in,13 patients (30.2%). None of the patients required transplacental anti-arrhythmic drug therapy and developed hydrops. Out of the total number of children, 37 (76.7%) were born after an urgent caesarean section, and 18 (41.8%) were born prematurely. Maternal systemic disease was present in 19 patients (44.1%), including maternal infection during pregnancy (n = 6), hypothyroidism (n = 5), preeclampsia (n = 3), intrahepatic cholestasis of pregnancy (n = 4), and gestational diabetes mellitus (n = 1). Time to initial control of tachycardia > 24 h was present in 12 patients (27.9%), and digitalis was used in 23 patients (53.4%).

CHD was present in 20 patients (46.5%), with complexity observed in 1 patient (2.3%) who had Ebstein anomaly. In the remaining 19 patients, CHD was simple and included atrial septal defect, patent ductus arteriosus, a combination of these two, or a ventricular septal defect. Systemic infection occurred in 35 patients (81.3%) during initial therapy, mainly due to neonatal pneumonia, with a few cases of necrotizing enterocolitis and suppurative encephalitis. Electrolyte disorders were present in 27 patients (62.7%), including hyperkalemia (n = 8), hypokalemia (n = 8), hypernatremia (n = 5), hypocalcemia (n = 21), either alone or in combination. Lab results showed that hypoproteinemia was found in 14 (32.5%) patients, anemia in 10 (23.2%) patients, acidosis in 8 (18.6%) patients, and hypoxemia in 20 (46.5%) patients. Additionally, the average plasma BNP level was significantly higher than normal.

**Fig. 1:**
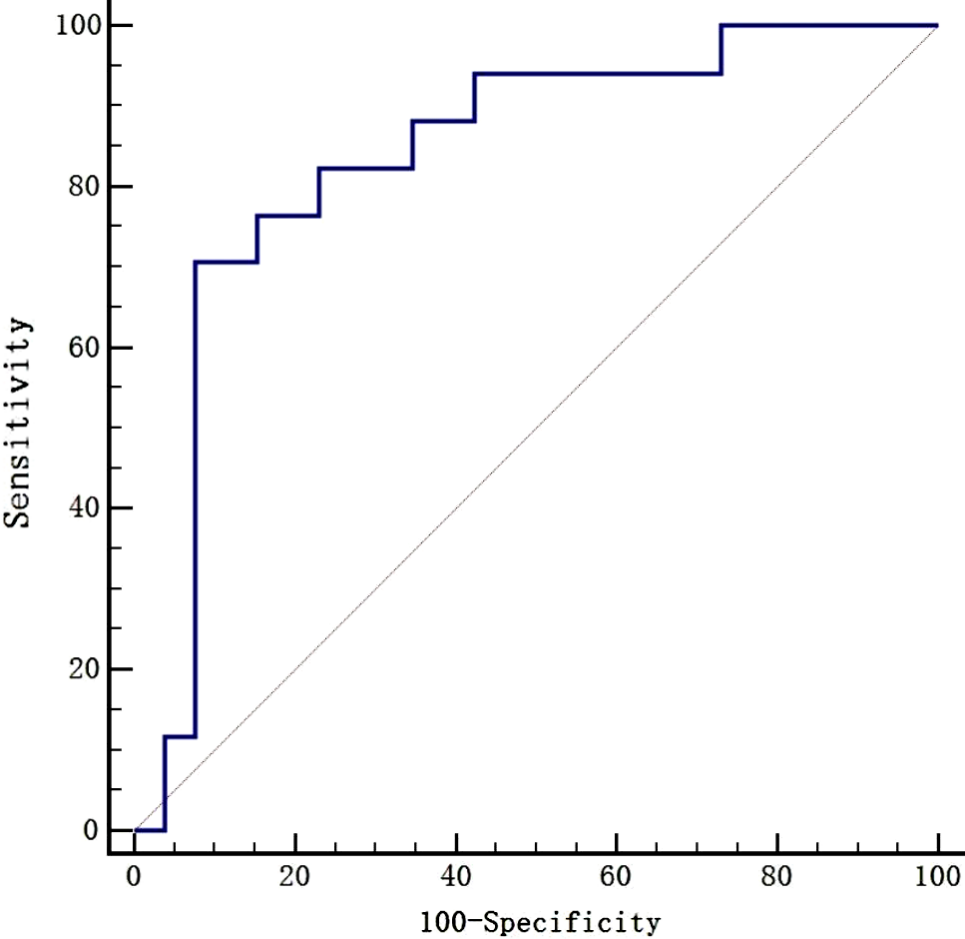
ROC curve analysis of BNP in predicting AHF secondary to SVT in neonates. SVT: supraventricular tachycardia; AHF: acute heart failure; BNP: B-type natriuretic peptide

Univariate logistic analysis was performed on the aforementioned variables to evaluate their correlation with AHF secondary to SVT (Table 1). Time to initial control of tachycardia > 24 h (OR 4.89, 95% CI 1.17 ~ 20.41), hyperkalemia (OR 6.55, 95% CI 1.14 ~ 37.75), anemia (OR 5.37, 95% CI 1.15 ~ 25.11), and plasma BNP level (OR 1.001, 95% CI 1.000 ~ 1.001) were significant in univariate analysis (all *P* < 0.05). Other variables did not show any association (all *P >* 0.05). The ROC curves for using BNP to predict AHF secondary to SVT were analyzed (Fig. 1), the AUC was demonstrated to be 0.80. According to the maximum Youden index, the optimal cutoff points for BNP were 2460.5 pg/ml, yielding a specificity of 84.6% and a sensitivity of 70.6%. A multivariate logistic regression analysis was conducted for the model, consisting of the four significant variables mentioned above. The analysis revealed that BNP > 2460.5pg/ml was the only predictor for AHF secondary to SVT in neonates (OR 2.28, 95% CI 1.27 ~ 45.39) (Table 2).

**Table 1:** Data of the study subjects and univariate logistic regression analysis for AHF secondary to SVT in neonates

	SVT with AHF (n = 17)	SVT without AHF (n = 26)	OR	95% CI	*P* value
**Female gender, n (%)**	9(52.9)	17(65.4)	1.68	0.48–5.85	0.42
**Gestational age (wk)**	37.2 ± 2.9	36.4 ± 3.2	1.09	0.88–1.36	0.41
**Birth weight (g)**	3018.5 ± 717.8	2997.5 ± 789.5	1.0	0.99-1.00	0.93
**Age at diagnosis (d)**	9.29 ± 8.75	8.69 ± 11.37	1.01	0.95–1.07	0.85
**Prematurity, n (%)**	6(35.3)	12(46.2)	0.64	0.18–2.24	0.48
**Birth asphyxia, n (%)**	2(11.8)	1(3.8)	3.33	0.28–39.98	0.34
**Urgent cesarean rate, n (%)**	13(76.5)	20(76.9)	0.98	0.23–4.14	0.97
**Fetal distress, n (%)**	5(29.4)	8(30.8)	0.94	0.25–3.56	0.93
**Prenatal tachyarrhythmia, n (%)**	3(17.6)	9(34.6)	0.41	0.09–1.79	0.23
**Maternal systemic disease,n (%)**	8(47.1)	11(42.3)	1.21	0.35–4.15	0.76
**Time to initial control of tachycardia (h)**	18.1 ± 9.7	13.1 ± 8.9	1.06	0.99–1.14	0.09
**Time to initial control of tachycardia > 24 h, n (%)**	8(47.1)	4(15.4)	4.89	1.17–20.41	0.03*
**Comorbid conditions, n (%)**					
Congenital heart disease	9(52.9)	11(42.3)	1.53	0.45–5.25	0.49
Acyanotic lesions					
ASD	4(23.5)	1(3.8)	7.69	0.78–76.08	0.08
PDA	3(17.6)	6(23.1)	0.71	0.15–3.35	0.67
ASD + VSD	0	1(3.8)	-	-	-
PDA + ASD	0	3(11.5)	-	-	-
ASD + VSD + PDA	1(5.9)	0	-	-	-
Ebstein anomaly	1(5.9)	0	-	-	-
Electrolyte disorders, n (%)	11(64.7)	16(61.5)	1.15	0.32–4.08	0.83
Hyperkalemia	6(35.3)	2(7.7)	6.55	1.14–37.75	0.04*
Hypokalemia	3(17.6)	5(19.2)	0.9	0.19–4.38	0.89
Hypernatremia	2(11.8)	3(11.5)	1.02	0.15–6.86	0.98
Hypocalcemia	7(41.2)	14(53.8)	0.6	0.174–2.065	0.42
Other systemic diseases, n (%)	16(94.9)	19(73.1)	5.89	0.65–53.11	0.11
Hypoproteinemia,n (%)	6(35.3)	8(30.8)	1.23	0.34–4.49	0.76
Anemia,n (%)	7(41.2)	3(11.5)	5.37	1.15–25.11	0.03*
Acidosis,n (%)	5(29.4)	3(11.5)	3.19	0.65–15.70	0.15
Hypoxemia,n (%)	10(58.8)	10(38.5)	2.29	0.66–7.96	0.19
**BNP(pg/ml)**	3371.87 ± 1749.19	1557.68 ± 1816.17	1.001	1.000-1.001	0.01*
**Digitalis**, n (%)	10(58.8)	13(50.0)	1.43	0.42–4.91	0.57

*Statistically significant (*P* < 0.05)

SVT: supraventricular tachycardia; AHF: acute heart failure; ASD: atrial septal defect; PDA: patent ductus arteriosus; VSD: ventricular septal defect; BNP: B-type natriuretic peptide

**Table 2:** Multivariate logistic regression model for AHF secondary to SVT in neonates

Indicator	*P*	*OR*	*95%CI*
Hyperkalemia	0.52	2.04	0.24 ~ 19.43
Anemia	0.35	2.59	0.35 ~ 19.43
Time to initial control of tachycardia > 24 h	0.84	1.22	0.17 ~ 8.49
BNP > 2460.5pg/ml	0.03	2.28	1.27 ~ 45.39

SVT: supraventricular tachycardia; AHF: heart failure; BNP: B-type natriuretic peptide

**Fig. 2:**
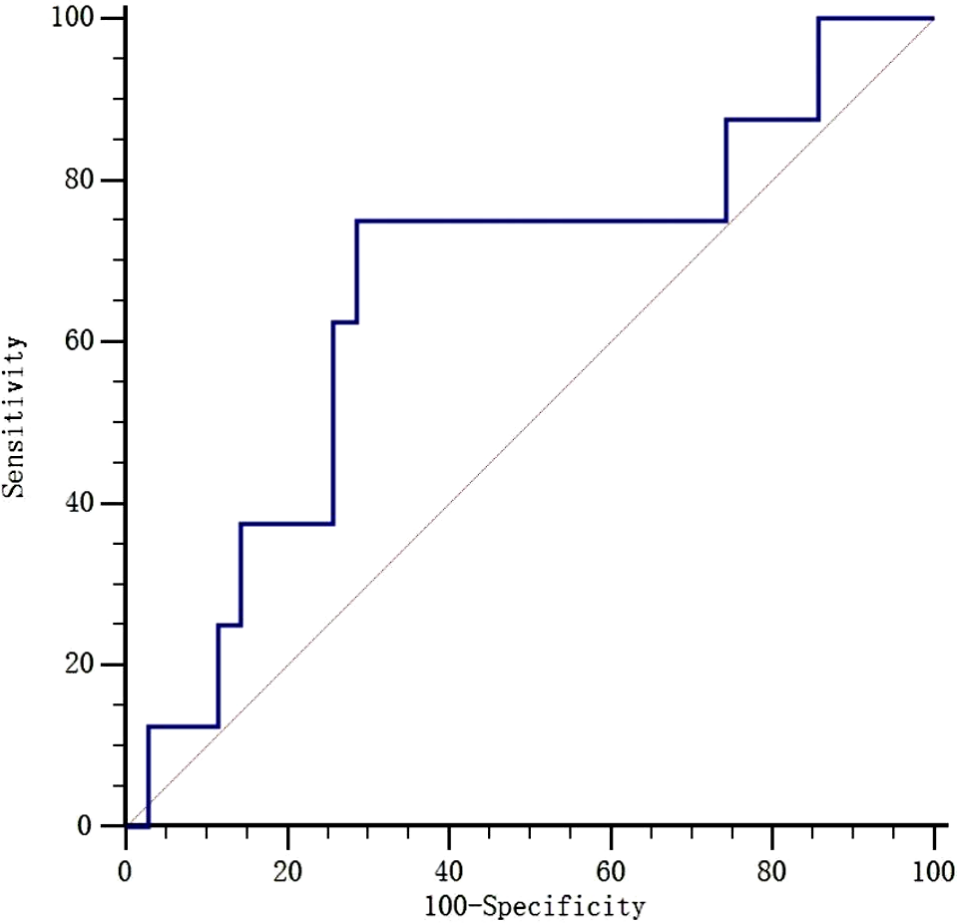
ROC curve analysis of BNP in predicting a demand of second line therapy. SVT: supraventricular tachycardia; AHF: acute heart failure; BNP: B-type natriuretic peptide

Anti-arrhythmic therapy for newborns with SVT, from initiation to termination, is outlined in Table 3. As a first-line treatment, standalone vagal maneuver achieved success in 10 patients (23.2%), while intravenous adenosine, digitalis and esmolol alone or in combination were successful in 25 patients (58.1%). Second line therapies such as intravenous propafenone, amiodarone, or DC cardioversion were required in 8 cases (18.6%) to ultimately terminate the tachycardia. The ROC curves for using BNP to predict the demand for second-line therapy were analyzed (Fig. 2). The AUC was found to be 0.66, and the optimal cutoff points for BNP were 2460.5 pg/ml, yielding a specificity of 71.4% and sensitivity of 75.0%. Patients with a BNP level of > 2460.5pg/ml had a higher demand for second-line therapy to control SVT compared to those with a BNP level of ≤ 2460.5 pg/ml (37.5% versus 7.4%, *P* = 0.04) (Table 4).

**Table 3:** Anti-arrhythmic therapy received from initiation to termination of supraventricular tachycardia

Anti-arrhythmic therapy	Total number of the patients (N = 43)	SVT with AHF (N = 17)	SVT without AHF (N = 26)
Vagal maneuver	10	2	8
Adenosine	6	1	4
Digitalis	6	2	5
Esmolol	1	0	1
Adenosine + digitalis	9	5	4
Digitalis + esmolol	2	0	2
Adenosine + digitalis + esmolol	1	0	1
DC cardioversion	1	1	0
Adenosine + DC cardioversion	2	2	0
Digitalis + propafenone	1	0	1
Adenosine + digitalis + amiodarone	2	2	0
Esmolol + amiodarone	1	1	0
Adenosine + digitalis + propafenone + DC cardioversion	1	1	0

Vagal maneuver was used in all objects, only valid terminations listed separately on the first line; +: used to represent sequential links; SVT: paroxysmalsupraventricular tachycardia; AHF: acute heart failure

**Table 4:** Predictive value of BNP at cut-off values for a demand of second line therapy

Treatment	BNP > 2460.5pg/ml	BNP ≤ 2460.5pg/ml	*X* ^***2***^	*P*
First line therapy (n, %)	10 (62.5%)	25 (92.6%)	5.89	0.04
Second line therapy (n, %)	6 (37.5%)	2 (7.4%)		

Vagal maneuver, adenosine, esmolol and digitalis are defined as first line therapy; DC cardioversion, propafenone, and amiodarone are defined as second line therapy; BNP: B-type natriuretic peptide

## Discussion

Supraventricular tachycardia (SVT) stands as the most common sustained arrhythmia in the neonatal age group, with an estimated incidence of infants as 0.25 per 1000 in infants and 0.06 per 1000 patients younger than one month [16]. AHF occurrs more commonly in 35% of patients under 4 months of age [17], a rate similar to the 39.5% incidence observed in our study. Due to the high tolerance and mild symptoms of neonates with supraventricular tachycardia (SVT) in the first 12–24 h, SVT often goes unnoticed for an extended period [18], resulting in frequent episodes of acute heart failure. Identifying risk factors contributing to the occurrence of AHF would assist in early detection and preventing decompensation.

In this study, we found that a longer duration of SVT— time to initial control of tachycardia > 24 h — could increase the risk of AHF. This finding is consistent with the study by Nadas et al., which reported a 19% incidence of AHF if tachycardia continued for 30 h, and a 50% incidence of AHF if tachycardia lasted for 48 h [19]. Comorbid conditions such as an inflammatory state, hypoxia, acidosis and electrolyte imbalance may trigger SVT and result in hemodynamic instability [20, 21]. In our study, hyperkalemia and anemia were identified as risk factors for AHF in SVT. B-type natriuretic peptide (BNP), a cardiac peptide released by the heart ventricles in response to changes in the ventricular pressure and/or volume, has been reported to be associated with AHF in children from other causes such as CHD [22]. Salas et al. have found that an increase in BNP levels, measured in critically ill neonates requiring assisted mechanical ventilation, may predict hemodynamic changes and a poor prognosis [23]. BNP in our study was identified to be a risk factor for AHF in neonates secondary to SVT.

Structural heart disease contributes to cardiovascular collapse during a tachycardia episode [24], but our study did not identify it as a risk factor. This may be because the majority of subjects had simple congenital heart diseases with small shunt volumes, while other studies involved large shunt volumes or complex congenital heart disease. As cardiac pump reserve function is limited, especially in immature infants, a fast heart rate can lead to a declined cardiac output. Prenatal history, prematurity, intrauterine tachycardia and urgent caesarean section are considered indicators of decreased fetal circulation and may be associated with unfavorable clinical outcome in neonates with SVT [25, 26]. However, we didn’t find these results. Perhaps the condition of the newborns in our study is not as serious. Lower body weight and younger age were reported to be associated with a fatal or near-fatal outcome in infant with SVT [8]. However, in our study, this association does not appear to be linked to AHF, potentially due to the inclusion of subjects with similar age and weight in each group.

Indeed, BNP is rarely used as a biomarker in newborns, because it can be affected by extra-cardiac conditions such as anemia, severe infections. It can also be influenced by certain prenatal and postnatal factors, such as mothers with type 1 diabetes, prematurity, cesarean Sect. [27]. Reeves et al. observed an extremely high level of approximately 20,000 pg/mL of N-terminal pro-brain natriuretic peptide (NT-proBNP), which originates from the breakdown of BNP, in 3 neonates with decompensated SVT [28]. This finding indicates the potential of plasma BNP in predicting AHF secondary to SVT in neonates. In our study, the value of BNP for predicting AHF was found to be 2460.5pg/ml. That is significantly higher than 758.7 pg/mL ~ 741.4 pg/mL at the 97.5th percentile in normal infants aged from 0-30d, as reported by Cantinotti et al. [27]. Furthermore, among the associated risk factors, BNP > 2460.5pg/ml was identified as an independent predictor. This indicates that the incidence of AHF secondary to SVT is not only influenced by the tachycardia itself, but also by various other factors related to the overall cardiovascular status. Specifically, BNP may accurately reflect the overall situation.

In addition to early intervention based on the type of tachycardia, it is crucial to have an effective treatment option to promptly terminate SVT and prevent decompensation. Digitalis was the most commonly used first-line drug in our study. However, it does not appear to be superior to other first-line drugs in preventing HF. Although the combination of positive inotropic activity with negative chronotropic effects has been shown to reduce hospital admissions in heart failure [29], esmolol appears to be more effective in preventing AHF. Our findings indicate that esmolol successfully terminated SVT without developing HF in 4 neonates, including those who did not respond to digitalis. This suggests that the positive inotropic effect may not be fully advantageous when dealing with tachycardia with preserved ejection fraction. In this case, esmolol alone or in combination with digitalis may be more effective in controlling elevated haemodynamic parameters in patients with SVT, as reported [30].

Till now, cardiologists still face a dilemma in balancing efficiency and safety when terminating acute recurrent and persistent SVT in neonates. Compared to first-line treatment, second-line therapy has attractive efficacy in case of refractory SVT. However, it is considered a reserved option due to the reported relatively high incidence of systemic adverse effects. These effects include propafenone-induced cardiac arrest, amiodarone-induced hypothyroidism and pulmonary fibrosis, cardiac depression caused by DC cardioversion, and the potential for pro-arrhythmia [2, 15]. However, some authors advocate that amiodarone and propafenone are equally safe and effective when used with monitoring as the first-choice drugs, especially in infancy [31–33]. In this study, 8 newborns who developed AHF were successfully treated with second-line therapy to terminated the prolonged SVT without any adverse effects. We also observed that neonates with a BNP > 2460.5pg/ml had a higher likelihood of requiring second-line therapy to control SVT. The equal value of BNP in predicting AHF and anti-arrhythmic treatment may not be coincidental. That’s exactly what Bjeloševič et al. reported: heart failure is a possible predictor of arrhythmia persistence. The need for ablation and mortality rate are reduced by the common use of amiodarone and propafenone in terminating arrhythmia [34]. Though the diagnostic accuracy is not significant, further study is warranted to explore the value of BNP in monitoring and predicting treatment response.

This study has some limitations. First, the sample size of this study is small, and we cannot further subdivide the research subjects, such as by age. Second, the diagnosis of heart failure is made using ROSS score, which is partly subjective. Third, the selection of anti-arrhythmic drugs and doses was based on the preference of attending physician. This circumstance made it impossible to study and characterize a clearly defined therapy protocol. Fourth, the retrospective study design hindered the assessment of adverse effects of anti-arrhythmic treatment. None of these neonates exhibited significant adverse reactions. Therefore, the definition of relatively higher risk anti-arrhythmic treatment is based on previous literature reports. Finally, there are many factors that influence BNP levels, which should be considered in conjunction with the clinical condition of children when applying it. Despite these limitations, we believe that our data about neonatal SVT can be valuable for neonatologists. Further multicenter prospective studies are needed to confirm our findings.

## Conclusions

Our study illustrates the neonates with a plasma level of BNP > 2460.5pg/ml have an increased the risk of AHF after the onset of SVT. These neonates may require a second-line anti-arrhythmic treatment to expedite the termination of SVT and prevent decompensation.

## Data Availability

The datasets used and analyzed during the current study are not publicly available due to limitations of ethical approval involving the patient data and anonymity but are available from the corresponding author on reasonable request.

## Abbreviations

SVT: supraventricular tachycardia
AHF: acute heart failure
BNP: B-type natriuretic peptide
CHD: congenital heart disease
